# Design of Environmental-Friendly Carbon-Based Catalysts for Efficient Advanced Oxidation Processes

**DOI:** 10.3390/ma17112750

**Published:** 2024-06-05

**Authors:** Xinru Xu, Guochen Kuang, Xiao Jiang, Shuoming Wei, Haiyuan Wang, Zhen Zhang

**Affiliations:** 1 Key Laboratory of Organic Integrated Circuit, Ministry of Education & Tianjin Key Laboratory of Molecular Optoelectronic Sciences, Department of Chemistry, School of Science, Tianjin University, Tianjin 300072, China; xuxinru789_@tju.edu.cn (X.X.); gckuang@tju.edu.cn (G.K.); xiaojiang@tju.edu.cn (X.J.); active_pp@163.com (S.W.); 2 National Demonstration Center for Chemistry and Chemical Engineering Education, Tianjin University, Tianjin 300350, China

**Keywords:** advanced oxidation processes, rhodamine B, peroxymonosulfate, ^1^O_2_

## Abstract

Advanced oxidation processes (AOPs) represent one of the most promising strategies to generate highly reactive species to deal with organic dye-contaminated water. However, developing green and cost-effective catalysts is still a long-term goal for the wide practical application of AOPs. Herein, we demonstrated doping cobalt in porous carbon to efficiently catalyze the oxidation of the typically persistent organic pollutant rhodamine B, via multiple reactive species through the activation of peroxymonosulfate (PMS). The catalysts were prepared by facile pyrolysis of nanocomposites with a core of cobalt-loaded silica and a shell of phenolic resin (Co-C/SiO_2_). It showed that the produced ^1^O_2_ could effectively attack the electron-rich functional groups in rhodamine B, promoting its molecular chain breakage and accelerating its oxidative degradation reaction with reactive oxygen-containing radicals. The optimized Co-C/SiO_2_ catalyst exhibits impressive catalytic performance, with a degradation rate of rhodamine B up to 96.7% in 14 min and a reaction rate constant (*k*) as high as 0.2271 min^−1^, which suggested promising potential for its practical application.

## 1. Introduction

With the development of industry, water pollution poses serious threats to human health and well-being, and the organic pollution of freshwater resources has become one of the most serious problems to solve. In particular, industrial dye is the main contributor to the pollution of water, rivers, and groundwater. It not only threatens the surrounding ecosystem by preventing light from reaching the surface, hindering plant photosynthesis, and lowering the oxygen content of the water leading to the death of aquatic organisms and plants, but also causes respiratory injury, tissue necrosis, reproductive system harm, and even genetic mutations in humans [1]. It has been reported that the worldwide production of rhodamine dyes alone will reach USD 232.5 million by 2027.

To achieve the goal of cleansing the water, many methods for removing organic pollutants have been examined, such as physical adsorption [2,3,4], biological methods [5,6], and advanced oxidation processes (AOPs). Among them, the AOPs have been widely proposed for the degradation of recalcitrant organic contaminants by the highly reactive species generated from oxidizing agents, such as H_2_O_2_ [7,8], peroxymonosulfate (PMS) [9,10,11,12], and peroxodisulfate (PDS) [13,14,15,16,17]. Over the past decades, various methods have been explored to activate oxidizer precursors, such as ultraviolet [18], ultrasound [19], thermal [20], and inorganic and organic activation [21]. Notably, heterogeneous catalysts, especially transition metal-based materials, have been extensively studied as heterogeneous catalysts in AOPs [22] owing to their lower energy requirements and easy scale-up for practical applications. However, despite the number of exciting advances achieved using heterogeneous catalysts for oxidizing agent activation, the practical application of such AOPs is often limited. It has been reported that the general metal ion leaching [23] and low catalytic activity [24,25,26] are the main reasons for the limited performance of AOPs. Therefore, the development of green and cost-effective catalysts is essential to realize the wide practical application of AOPs in environmental remediation.

The key to AOPs is the use of highly reactive oxygen species (ROS), including free radicals (•OH, SO_4_^−▪^, O_2_^−▪^, etc.) and strongly oxidizing non-free radicals (e.g., ^1^O_2_, O_3_, etc.), to degrade and even completely mineralize organic pollutants into CO_2_ and H_2_O through complex electron exchange processes [27]. It is clear that the oxidizing species involved in AOPs determine the degradation and transformation behavior of organic matter, such as degradation rates, pathways, and by-products. For example, the reactive oxygen-containing radicals of •OH, with high oxidation potential (*E*_0_ = 1.8–2.7 V), are considered as some of the most powerful oxidants, which can react with numerous organic compounds [28]. However, such radical-based AOPs are less effective in degrading certain persistent organic pollutants (POPs) like the highly toxic halogenated phenols. The oxidation potential of ^1^O_2_ is relatively mild (*E*_0_ = 1.4–2.2 V), but it tends to attack the electron-rich organic compound [29,30]. Therefore, taking into account the degradation characteristics of ROS in AOP as well as the structural properties of specific molecules to design catalysts is expected to be effective in improving the advanced oxidative degradation of POPs.

In order to develop green and cost-effective catalysts for AOPs to deal with the ever-growing pollution of industrial dye and to purify freshwater resources, in this paper, we design catalysts based on the structural characteristics of pollutant molecules. Typically, for the electron-rich persistent organic pollutant rhodamine B (RhB), we synthesized a catalyst with cobalt doped in porous carbon for its efficient catalytic degradation via the activation of PMS. The catalysts were prepared by facile pyrolysis of nanocomposites with a core of cobalt-loaded silica and a shell of phenolic resin (Co-C/SiO_2_). Tiny amounts of Co ions that are effectively anchored by the presence of -OH in phenolic resins (RF) inhibit sintering during calcination and leaking during organic removal. Radical quenching experiments indicate the generation of active species like •OH, SO_4_^−▪^, and ^1^O_2_ in the AOPs catalyzed on Co-C/SiO_2_ catalysts. Since ^1^O_2_ can efficiently attack the electron-rich functional groups in rhodamine, the chain of rhodamine molecules is rapidly broken, accelerating its oxidative degradation. The optimized Co-C/SiO_2_ catalyst exhibits impressive catalytic performance, with a degradation rate of rhodamine B up to 96.7% in 14 min and a reaction rate constant (*k*) as high as 0.2271 min^−1^. Our study details useful insights into the design of catalysts used in AOPs based on the characteristics of organic molecules for the efficient and sustainable remediation of organic pollutants.

## 2. Materials and Methods

### 2.1. Chemicals and Materials

Tetraethyl silicate (98%), ammonia (25–28%), ethanol (≤0.3%), anhydrous sodium sulfate (99%) and rhodamine B (MW: 479.01) were obtained from Shanghai Aladdin Biochemical Technology Co., Ltd. (Shanghai, China). Cobalt chloride hexahydrate (99%) was obtained from Annaiji 3A Chemistry Co., Ltd. (Shanghai, China). Polyvinylpyrrolidone (MW: 40,000) was obtained from Shanghai Yuanye Co., Ltd. (Shanghai, China). Resorcinol (99%), tert-butanol (99.5%) and furfuryl alcohol (98%) were obtained from Shanghai Energy Chemical Co., Ltd. (Shanghai, China). Formaldehyde (37.0–40.0%) was obtained from Tianjin Yuanli Chemical Co., Ltd. (Tianjin, China). Potassium permonosulfate complex salt (47%) and Sulfamethoxazole (MW: 253.28) were obtained from Xiensi reagent Co., Ltd. (Tianjin, China). Bisphenol A (MW: 228.29) was obtained from Macklin Chemical Co., Ltd. (Shanghai, China). Methyl orange (MW: 327.33) and sodium carbonate (≥99.8%) were obtained from Macklin Chemical Reagent Co., Ltd. (Shanghai, China). Methanol (≥99.9%) was obtained from Shanghai Mackin Biochemical Co., Ltd. (Shanghai, China) Potassium monobasic phosphate (99%) and p-benzoquinone (99%) were obtained from Shanghai Merrell Biochemical Technology Co., Ltd. (Shanghai, China) Sodium chloride (≥99.5%) was obtained from Tianjin Jiangtian Chemical Co., Ltd. (Tianjin, China). All chemicals were at reagent or above purity and were used without further purification. Deionized water was used in all experiments.

### 2.2. Synthesis of Co/SiO_2_

Colloidal silica nanospheres were prepared through a modified Stöber method [31]. In a typical synthesis for ~250 nm particles, 2.25 mL of tetraethyl silicate (TEOS) was added into a mixture containing 36.75 mL of ethanol, 7.5 mL of water, and 3.5 mL of aqueous solution of ammonia (28%). After stirring for 2 h at room temperature, the silica particles were collected by centrifugation, washed with ethanol and water, and then re-dispersed in 12 mL of water. Then, 2 mL of the as-prepared silica spheres was mixed with 22 mL of water and 1 mL of 50 mM CoCl_2_·6H_2_O and kept overnight under stirring. Then the samples were collected by centrifugation, washed several times with water, and labeled as Co/SiO_2_.

### 2.3. Synthesis of Co-C/SiO_2_

A layer of resorcinol-formaldehyde (RF) was coated on the surface of the formed Co/SiO_2_ according to a modified version of a reported procedure [32]. In a typical process, the as-prepared Co/SiO_2_ particles were mixed with 46 mL of water and 4 mL of polyvinylpyrrolidone (PVP) aqueous solution (5 wt%). After stirring overnight, the above Co/SiO_2_ particles were collected by centrifugation, re-dispersed in 56 mL of water, and then combined with resorcinol (0.10 g), formaldehyde aqueous solution (37%, 0.14 mL), and 1 mL of dilute ammonia aqueous solution (2.8%). After heating the solution at 60 °C for two hours and then 80 °C for an hour, particles with RF were collected by centrifugation, washed with ethanol and water, and subsequently freeze-dried at −50 °C in a freeze dryer overnight. The obtained samples were carbonized in a tube furnace under a flowing nitrogen atmosphere at a high temperature of 700 °C for 2 h, with a rate of temperature rise of 5 °C/min^−1^, and labeled as Co-C/SiO_2_.

### 2.4. Characterizations

The morphology and particle size of the nanospheres were characterized by scanning electron microscopy (SEM, SU8010, Hitachi, Tokyo, Japan) and transmission electron microscopy (TEM, JEOL-2100 F, JEOL, Tokyo, Japan). Elemental mapping and energy-dispersive spectrometry (EDS) were performed on a JEM-3010. The element valence states were conducted by the X-ray photoelectron spectroscopy (XPS, ESCALAB 250Xi, Thermo Fisher, Waltham, MA, USA). X-ray powder diffraction (XRD) was collected on an X-ray diffraction instrument with Cu-Ka radiation from 5° to 89° (RIGAKU SMARTLAB9KW, Rigaku, Tokyo, Japan). Ultraviolet-visible (UV-vis) absorption spectra were collected on a UV-visible spectrophotometer (UV-3600Plus, Shimadzu, kyoto, Japan). The Brunauer–Emmett–Teller (BET) surface area and porous structure were measured by nitrogen adsorption and desorption (N_2_ adsorption-desorption) at 77.3 K using a MicrotracBEL Inc. (Shanghai, China) model BELSOPR-max analyzer, and the samples were degassed at 300 °C for 8 h under vacuum (10^−^^5^ bar) before analysis.

### 2.5. Catalytic Activity

The catalyst degradation performance was conducted at 25 °C in a 100 mL reactor containing 25 mg/L RhB as an initial solution. In a typical experiment, 8 mg of catalyst was added into 40 mL of RhB solution and stirred for 30 min to establish the adsorption-desorption equilibrium. The reaction was initiated by adding 0.2 g/L of PMS aqueous solution. Next, 2 mL of the reaction solution was withdrawn at a certain time interval, filtered through a 0.22 µm filter membrane, and immediately quenched with 0.2 mL of methanol solution. For the cycling test, the catalyst was recycled after each run of the experiment with ethanol and deionized water and then recalcined at 700 °C under a N_2_ atmosphere. The concentration of RhB was analyzed by an ultraviolet-visible (UV-Vis) spectrometer at 554 nm wavelength. The concentration of other typical organic contaminants, including bisphenol A (BPA), methyl orange (MO), tetracycline (TC), and sulfamethoxazole (SMZ), were also analyzed by the UV-Vis spectrometer at the detection wavelengths of 278 nm, 465 nm, 357 nm, and 265 nm, respectively. The reaction rate was evaluated by a pseudo-first-order kinetics model (Equation (1)):

ln (*C*/*C*_0_) = − *kt* (1)

where *C*_0_ is the initial pollutant concentration, and *C* is the concentration at a certain time during the degradation process.

### 2.6. A Modified Kinetic Model (k-Value)

The improved kinetic model *k*-value (×10^−^^3^ min^−^^1^) was obtained by multiplying the reaction rate constant *k* (min^−^^1^) and the ratio of pollutant concentration *C*_p_ (mg/L) to catalyst concentration *C*_c_ (mg/L) (Equation (2)):

*k*-value = *k* × (*C*_p_/*C*_c_) (2)

## 3. Results and Discussion

### 3.1. Synthesis and Characterization of Co-C/SiO_2_ Catalyst

**Figure 1:**
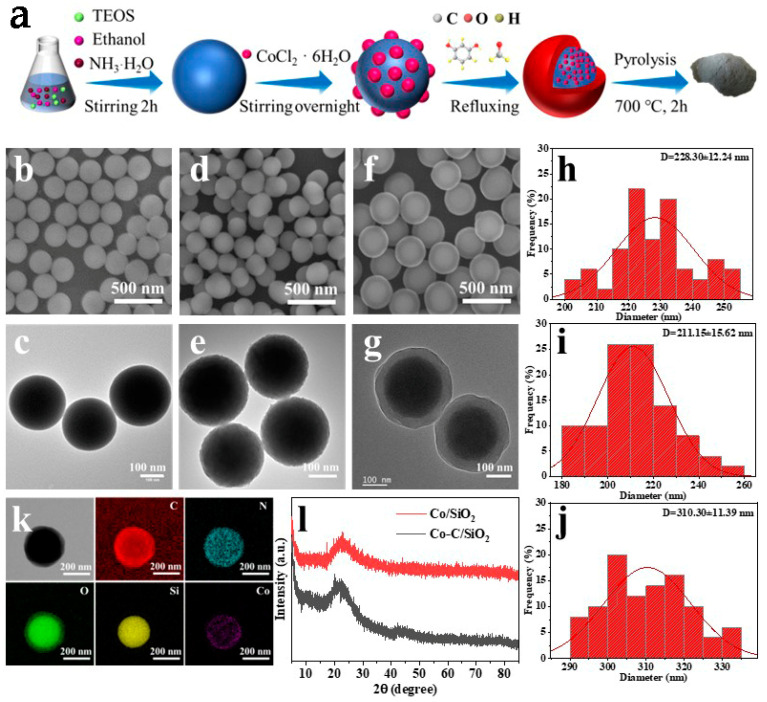
(**a**) Schematic illustration of the preparation process of the Co-C/SiO_2_ catalyst with core-shell structure; (**b**) SEM and (**c**) TEM of SiO_2_; (**d**) SEM and (**e**) TEM of Co/SiO_2_; (**f**) SEM and (**g**) TEM of Co-C/SiO_2_; the average size distribution diagram of (**h**) SiO_2_; (**i**) Co/SiO_2_ and (**j**) Co-C/SiO_2_; (**k**) EDS elemental mapping images of C, N, O, Si, Co in Co-C/SiO_2_.

A schematic illustration of the preparation process of the catalyst is shown in Figure 1a. The morphologies of samples obtained at each step of the reaction were characterized using scanning electron microscopy (SEM) and transmission electron microscopy (TEM). Both SEM (Figure 1b) and TEM (Figure 1c) demonstrated the relatively uniform size of silica nanospheres prepared by the modified Stöber method. It can be observed that the size of the silica nanospheres after cobalt loading did not increase significantly from Figure 1d, but their surface changed from smooth to rough, which demonstrates that the cobalt successfully loaded to form Co/SiO_2_ nanospheres (Figure 1e). It can also be observed from both SEM and TEM images that the Co/SiO_2_ nanospheres, after the reflux reaction, had formed a phenolic resin (RF) shell with a distinct core-shell structure (Figure 1f,g), thus forming Co-C/SiO_2_ nanospheres. In order to verify the distribution of the elements, EDS elemental mapping characterization was carried out, and a uniform distribution of the elements C, N, O, Si and Co was observed in the Co-C/SiO_2_ nanospheres (Figure 1k). The X-ray diffraction (XRD) was performed to understand the crystallinity of Co-C/SiO_2_ nanospheres, and the results showed that both Co/SiO_2_ and Co-C/SiO_2_ have the same diffraction peak position and amorphous structure (Figure 1l) demonstrating that RF did not contribute to the increase in crystallinity. In addition, to understand the specific surface area and pore size distribution of Co-C/SiO_2_ nanospheres, the N_2_ adsorption-desorption isotherm was determined by the Brunauer–Emmett–Teller (BET) method using the microphone device, and the results showed a type IV isotherm (Figure S1) and indicated the mesoporous structure. The Co-C/SiO_2_ presented a notable specific surface area of 256.44 m^2^/g, the total pore volume was 0.1942 cm^3^/g, and the average pore diameters were 3.0297 nm. Furthermore, comparing the BET of Co-C/SiO_2_ with that of other catalysts (Table S1), it was found that the higher specific surface area of the catalysts helped to provide more active sites to improve the degradation efficiency of RhB.

### 3.2. Catalytic Performance of Co-C/SiO_2_ in AOPs

**Figure 2:**
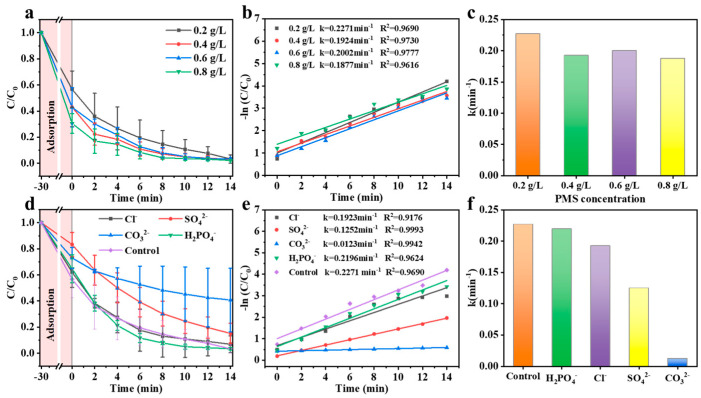
Effects of various factors on RhB removal in the Co-C/SiO_2_-PMS system. (**a**) Effect of PMS concentration; (**b**) the corresponding kinetics and (**c**) the corresponding degradation rate constants (*k*) of RhB; (**d**) effect of inorganic anions; (**e**) the corresponding kinetics and (**f**) the corresponding degradation rate constants (*k*) of RhB. Conditions: [Catalyst] = 0.2 g/L, [PMS] = 0.2 g/L, [RhB] = 25 mg/L, pH = 6.6, T = 25 °C.

It is widely acknowledged that PMS plays a crucial role in the Fenton-like degradation of organic compounds because it can directly generate SO_4_^•−^, known for its rapid degradation of contaminants [33]. Consequently, the influence of PMS concentration (0.2–0.8 g/L) on RhB degradation was investigated. Apparently, RhB can be almost completely degraded within 14 min as the concentration of PMS increases from 0.2 to 0.8 g/L (Figure 2a). The findings indicated that PMS may enhance the generation of free radicals. However, Figure 2b,c revealed that the reaction rate constant *k* peaks (0.2271 min^−^^1^) at a PMS dosage of 0.2 g/L. Thus, it means 0.2 g/L PMS will be enough for the degradation of RhB, making it the most cost-effective option.

To explore the potential of the Co-C/SiO_2_-PMS system in practical applications, the impact of inorganic anions (Cl^−^, SO_4_^2−^, CO_3_^2–^, H_2_PO_4_^−^, up to 10 mM) on the elimination of RhB was detected. Observing Figure 2d, it was inferred that Cl^−^ and H_2_PO_4_^−^ showed no clear impact on RhB degradation, yet there was a noticeable reduction in both reaction rates (Figure 2e,f). This is because Cl^-^ is capable of directly interacting with HSO_5_^−^ to consume a part of PMS and produce HOCl (Equation (3)) [34], while H_2_PO_4_^−^ can inhibit the generation of ROS (Equation (4)) [35]. Upon the introduction of SO_4_^2−^, the removal rate was significantly decreased, along with a decrease in the removal efficiency of RhB. The reason is that SO_4_^2−^ is capable of participating in ROS conversion and generating e^−^ (Equation (5)) [36]. There was a noticeable effect observed with CO_3_^2–^. The degradation efficiency of RhB decreased from 96.7% to 44.3% in the presence of 10 mM CO_3_^2–^ since CO_3_^2–^ can also effectively scavenge SO_4_^•−^ (Equation (6)) [35].

HSO_5_^−^ + Cl^−^ → SO_4_^2−^ + HOCl (3)

•OH + H_2_PO_4_^−^ → H_2_PO_4_^•^ + OH^−^  *k* = 2.0 × 10^4^ M^−1^s^−1^ (4)

SO_4_^•−^ + SO_4_^2−^ → S_2_O_8_^2−^ + e^−^  *k* = 5.0 × 10^8^ M^−1^s^−1^ (5)

SO_4_^•−^ + CO_3_^2–^ → CO_3_^•−^ + SO_4_^2−^  *k* = 6.1 × 10^6^ M^−1^s^−1^ (pH ≥ 11) (6)

**Figure 3:**
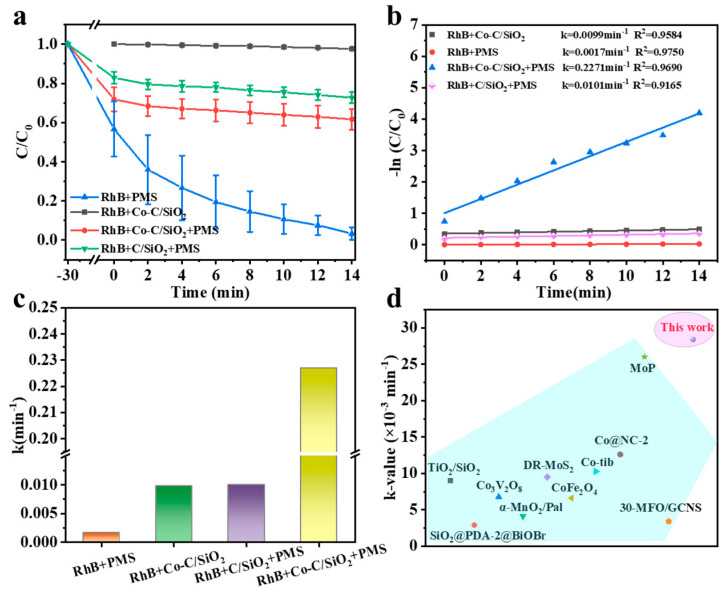
(**a**) RhB removal in different systems; (**b**) the corresponding kinetics and (**c**) the corresponding degradation rate constants (*k*) of RhB; (**d**) comparison of the Co-C/SiO_2_ catalyst with other Fenton-like catalysts. Conditions: [Catalyst] = 0.2 g/L, [PMS] = 0.2 g/L, [RhB] = 25 mg/L, pH = 6.6, T = 25 °C.

RhB degradation experiments were conducted to investigate the catalytic properties of Co-C/SiO_2_. Remarkably, the reaction system with Co-C/SiO_2_+PMS+RhB can almost completely degrade RhB within only 14 min, with the highest degradation efficiency of 96.7%, while single PMS and Co-C/SiO_2_ can only slightly degrade RhB, and their degradation efficiency levels were 2.37% and 38.3%, respectively (Figure 3a). In addition, the C/SiO_2_+PMS+RhB system degraded RhB only 29.7% within 14 min compared to the Co-C/SiO_2_+PMS+RhB system, highlighting the importance of Co species in enhancing RhB degradation efficiency (Figure 3a). Furthermore, the reaction rate constant (*k*) of Co-C/SiO_2_ is 0.2271 min^−^^1^, surpassing that of other related catalytic systems (Figure 3b,c), indicating the superior catalytic degradation performance of Co-C/SiO_2_. Comparing the *k*-value of Co-C/SiO_2_ and other Fenton-like catalysts, it was found that the fabricated catalysts had a higher *k*-value, which proved the efficient pollutant degradation ability of the Co-C/SiO_2_ catalyst (Figure 3d and Table 1).

**Figure 4:**
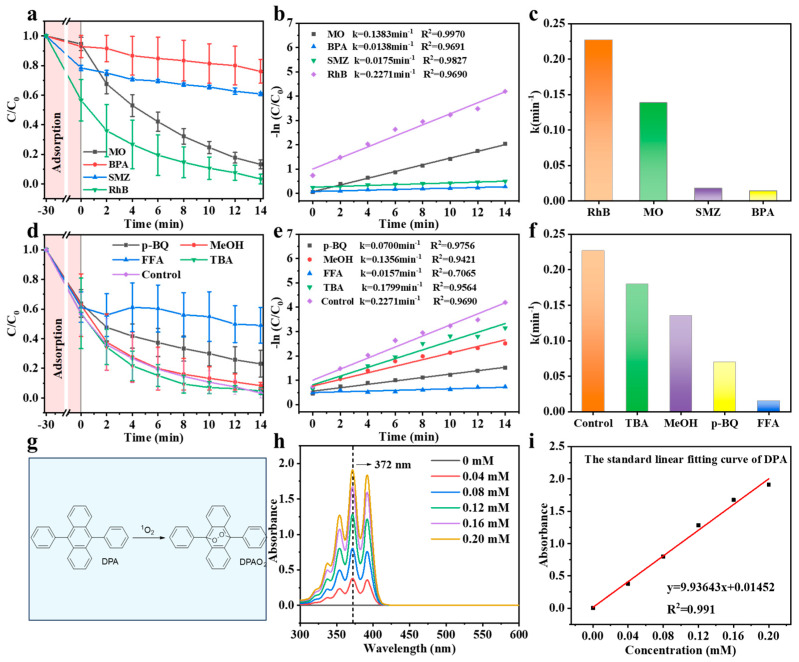
(**a**) Universality of Co-C/SiO_2_; (**b**) the corresponding kinetics and (**c**) the corresponding degradation rate constants (*k*); (**d**) RhB removal with different scavengers; (**e**) the corresponding kinetics and (**f**) the corresponding degradation rate constants (*k*) of RhB. Conditions: [Catalyst] = 0.2 g/L, [PMS] = 0.2 g/L, [RhB] = 25 mg/L, pH = 6.6, T = 25 °C; (**g**) diagram reaction of DPA with ^1^O_2_; (**h**) UV-vis absorption spectrum of DPA at different concentrations; (**i**) the fitting curve of concentration gradient of DPA at the absorption peak of 372 nm.

Subsequently, the potential for degradation of the Co-C/SiO_2_-PMS system was examined for other typical organic contaminants. For this purpose, methyl orange (MO), sulfamethoxazole (SMZ), and bisphenol A (BPA) were used as model contaminants for degradation by the Co-C/SiO_2_-PMS system. The results demonstrated that the Co-C/SiO_2_ system exhibited a relatively high catalytic degradation capacity for MO, achieving a degradation efficiency of up to 87% within 14 min (Figure 4a) and a reaction rate constant of *k* 0.1383 min^−^^1^ (Figure 4b,c). For SMZ and BPA, there was a certain degree of degradation observed within a short period of 14 min. Although the Co-C/SiO_2_-PMS system exhibits a different removal for each type of contaminant, the results prove that the Co-C/SiO_2_ catalyst exhibits a robust performance in the degradation of a diverse array of organic contaminants.

Previous studies have indicated that the PMS activation process is not solely mediated by free radical pathways but also by non-free radical pathways [47]. To examine the reactive oxygen species involved in the Co-C/SiO_2_-PMS system, the quenching experiments were performed by the addition of tert-butanol (TBA) to quench •OH [48], methanol (MeOH) to scavenge both •OH and SO_4_^•−^ [49], p-benzoquinone (p-BQ) to scavenge O_2_^•−^ [50], and furfuryl alcohol (FFA) to scavenge ^1^O_2_ [51], respectively. The results showed that the decomposition efficiency of RhB after the addition of TBA was 95.3%, which was comparable to that of the control group (96.7%), thereby indicating that •OH was nearly ineffective in the Co-C/SiO_2_-PMS system. The degradation efficiency of RhB with the addition of MeOH was 97.1%, with only a slight decrease compared to the control group, indicating that SO_4_^•−^ made a certain contribution to the degradation of RhB in the Co-C/SiO_2_-PMS system. However, when p-BQ was added into the Co-C/SiO_2_-PMS system, the degradation efficiency of RhB was severely reduced to 77.0%, which was 79.6% of the degradation efficiency of the control group (Figure 4d). Additionally, the reaction rate constant, *k*, decreased from 0.2271 min^−^^1^ of the control group to 0.0700 min^−^^1^ (Figure 4e,f). These results suggest that O_2_^•−^ exhibited a more pronounced catalytic ability for degrading RhB. It was unexpected that RhB was barely degraded when FFA was introduced into the Co-C/SiO_2_-PMS system. The remaining concentration of RhB decreased from 61.3% after the adsorption equilibrium to 49.0% at the end of the reaction. The degradation of RhB was observed to be only 12% after 14 min, with the reaction rate constant *k* reaching a minimum value of 0.0157 min^−^^1^ (Figure 4d–f). This indicates that the inhibition of the FFA was the most pronounced. This demonstrated that ^1^O_2_ is the main reactive oxygen species (ROS) generated in the catalytic degradation reaction. Therefore, it can be concluded that the following species are all involved in the RhB degradation process in the Co-C/SiO_2_-PMS system: •OH, SO_4_^•−^, O_2_^•−^ and ^1^O_2_. To further demonstrate the content of ^1^O_2_, 9,10-diphenanthraquinone dye (DPA) was chosen as the chemical trapping reagent to quantify the generation of ^1^O_2_ in the Co-C/SiO_2_-PMS system [52]; the reaction between DPA and ^1^O_2_ is shown in Figure 4g. Different concentrations of DPA solution were used to verify the presence of ^1^O_2_ (Figure 4h) and the fitting curve for DPA (Figure 4i) to calculate the content of ^1^O_2_ as 31.3 µM. A comparison between Co-C/SiO_2_ and other Fenton-like catalysts for ^1^O_2_ yield is shown in Table S3.

Based on the above results and analysis, the possible mechanisms of the Co-C/SiO_2_-PMS systems were proposed: (I) the free radical mechanism (•OH, SO_4_^•−^, O_2_^•−^) and (II) the non-free radical mechanism (^1^O_2_). Firstly, Co^2+^ reacted with PMS to produce SO_4_^•−^ and SO_4_^2−^ (Equation (7)). Subsequently, Co^3+^ could also be reduced into Co^2+^ by PMS (Equation (8)). The formed SO_4_^•−^ could participate in the generation of •OH (Equation (9)). These processes effectively promoted the redox cycle of Co^2+^/Co^3+^, thus enhancing the activation of PMS. Finally, the production of O_2_^•−^ occurred as a result of the self-decomposition of PMS under the condition of Co-C/SiO_2_ activation (Equation (10) and Equation (11)) and the interaction between •OH and H_2_O_2_ (Equations (12)–(14)). Next, the generated O_2_^•−^ could react with H_2_O and •OH to produce ^1^O_2_ (Equations (15) and (16)). Additionally, the formed SO_5_^•−^ could react with each other to produce ^1^O_2_ (Equation (17)). These processes contributed to maintaining the high activity of Co-C/SiO_2_ in PMS activation.

Co^2+^ + HSO_5_^−^ → Co^3+^ + SO_4_^•−^ + SO_4_^2−^ (7)

Co^3+^ + HSO_5_^−^ → Co^2+^ + SO_5_^•−^ + H^+^ (8)

SO_4_^•−^ + H_2_O → H^+^ + •OH + SO_4_^2−^ (9)

HSO_5_^−^ → H^+^ + SO_5_^2−^ (10)

SO_5_^2−^ + H_2_O → O_2_^•−^ + H^+^ + SO_4_^2−^ (11)

SO_5_^2−^ + H_2_O → H_2_O_2_ + SO_4_^2−^ (12)

•OH + 2H_2_O_2_ → HO_2_^•^ + H_2_O (13)

HO_2_^•^ → H^+^ + O_2_^•−^ (14)

O_2_^•−^ + 2H_2_O → ^1^O_2_ + H_2_O_2_ + 2OH^−^ (15)

O_2_^•−^ + •OH → ^1^O_2_ + OH^−^ (16)

SO_5_^•−^ + SO_5_^•−^ → ^1^O_2_ + 2SO_4_^•−^ (17)

**Figure 5:**
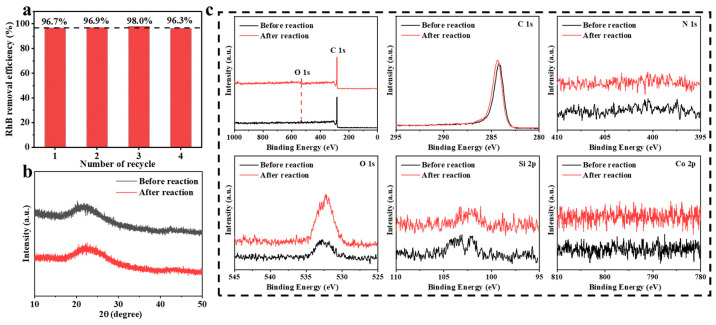
(**a**) Catalytic activity of Co-C/SiO_2_ for different cycle numbers. (**b**) XRD diffraction pattern of Co-C/SiO_2_ before and after the reaction. (**c**) XPS of Co-C/SiO_2_ before and after the reaction.

In order to investigate the stability of Co-C/SiO_2_, the degradation of RhB was monitored in four consecutive usages of Co-C/SiO_2_ under the same conditions. The reusability of Co-C/SiO_2_ is illustrated in Figure 5a, and the removal efficiency of RhB could be maintained at more than 96% for four cycles. Furthermore, there was no significant change in the XRD diffraction patterns of Co-C/SiO_2_ before and after the reaction (Figure 5b), indicating that the structure of Co-C/SiO_2_ remained stable throughout the catalytic process. Additionally, the chemical composition of the used Co-C/SiO_2_ was almost the same as that of the fresh Co-C/SiO_2_ with negligible changes in the XPS spectra (Figure 5c). The Co-C/SiO_2_ catalyst exhibited excellent reusability and stability towards RhB degradation in the presence of PMS.

## 4. Conclusions

The synthesized Co-C/SiO_2_ with a core-shell structure in this work exhibited high performance to activate PMS for the degradation of organic pollutants, with RhB (25 mg/L) completely removed within 14 min. Both SEM and TEM demonstrated that the Co-C/SiO_2_ system had an apparent core-shell structure. The radical quenching results demonstrated the existence of ^1^O_2_, SO_4_^•−^, •OH and O_2_^•−^ in the Co-C/SiO_2_-PMS system, whereas ^1^O_2_ played a primary role in the degradation of RhB. In addition, the Co-C/SiO_2_ system exhibited high stability and reusability, with over 96.7% of RhB degraded after four cycles. Overall, this work has provided an environmental-friendly carbon-based catalyst for the treatment of organic wastewater. Our study details the useful insights into the design of an environmental-friendly catalyst with high activity for green and efficient remediation of the environment.

## Figures and Tables

**Table 1:** Comparison of catalytic properties with recently reported Fenton-like catalysts for PMS/PS/H_2_O_2_ activation.

Catalyst (g/L)	PMS (g/L)	Pollutant (mg/L)	Removal Efficiency	*k* (min^−1^)	*k*-Value (×10^−3^ min^−1^)	Ref.
TiO_2_/SiO_2_ (0.02)	visible-light irradiation	RhB (10)	100%	0.018	9	[37]
SiO_2_@PDA-2@BiOBr (0.25)	visible-light irradiation	RhB (15)	96%	0.0487	2.9	[38]
Co_3_V_2_O_8_ (0.2)	PS (25 mM)	TC (50)	87.1%	0.0271	6.775	[39]
α-MnO_2_/Pal (0.1)	PMS (0.1)	RhB (20)	100%	0.02041	4.1	[40]
DR-MoS_2_ (0.1)	PMS (3.25 mM)	RhB (10)	92.1%	0.09533	9.5	[41]
CoFe_2_O_4_ (0.3)	PMS (7 mM)	RhB (20)	100%	0.0986	6.6	[42]
Co-tib (0.2)	PMS (0.15 mM)	RhB (10)	100%	0.2053	10.27	[43]
Co@NC-2 (0.4)	SO_3_^2−^ (5 mM)	MO (20)	100%	0.25232	12.62	[44]
MoP (0.1)	H_2_O_2_ (2.0 mM)	DCF (20)	100%	0.13	26	[45]
30-MFO/GCNS (0.2)	PMS (2)	SMX (5)	100%	0.1363	3.41	[46]
Co-C/SiO_2_ (0.2)	PMS (0.2)	RhB (25)	96.7%	0.2271	28.4	This work

## Data Availability

Data are contained within the article.

## Supplementary Materials

The following supporting information can be downloaded at: https://www.mdpi.com/article/10.3390/ma17112750/s1, Figure S1: N_2_ adsorption-desorption isotherm plot of Co-C/SiO_2_; Table S1: BET comparison between Co-C/SiO_2_ and other catalysts; Table S2: Comparison of the binding energy and content of each element of Co-C/SiO_2_ before and after the reaction; Table S3: Comparison between Co-C/SiO_2_ and other Fenton-like catalysts for ^1^O_2_ yield. References [52,53,54,55,56,57,58] are cited in the supplementary materials.
