# Supplementary material for: Design of Environmental-Friendly Carbon-Based Catalysts for Efficient Advanced Oxidation Processes
Source: Materials (Basel). 2024 Jun 5;17(11):2750. doi: 10.3390/ma17112750 (PMC11173702; doi:10.3390/ma17112750)
Supplement: Supplementary file 1 [file materials-17-02750-s001.zip › materials-3013232-supplementary.pdf]

Supporting Information

Design of environmental-friendly carbon-based catalysts for efficient advanced oxidation processes

Xinru Xu [1], Guochen Kuang [1], Xiao Jiang [1], Shuoming Wei [1], Haiyuan Wang [2,*] and Zhen Zhang [1,*]

[1] Key Laboratory of Organic Integrated Circuit, Ministry of Education & Tianjin Key Laboratory of Molecular Optoelectronic Sciences, Department of Chemistry, School of Science, Tianjin University, Tianjin 300072, China; xuxinru789_@tju.edu.cn (X.R.X); gckuang@tju.edu.cn (G.C.K.); xiaojiang@tju.edu.cn (X.J.) 313927002@qq.com (S.M.W)
[2] National Demonstration Center for Chemistry and Chemical Engineering Education, Tianjin University, Tianjin 300350, China; haiyuan.wang@tju.edu.cn (H.Y.W.)
* Correspondence: zhzhen@tju.edu.cn; haiyuan.wang@tju.edu.cn.

Supplementary Figures and Tables

Figure S1. N_2 adsorption-desorption isotherm plot of Co-C/SiO_2.
Table S1. BET comparison between Co-C/SiO_2 and other catalysts.
Table S2. Comparison of the binding energy and content of each element of Co-C/SiO_2 before and after the reaction.
Table S3. Comparison between Co-C/SiO_2 and other Fenton-like catalysts for $^1O_2$ yield.

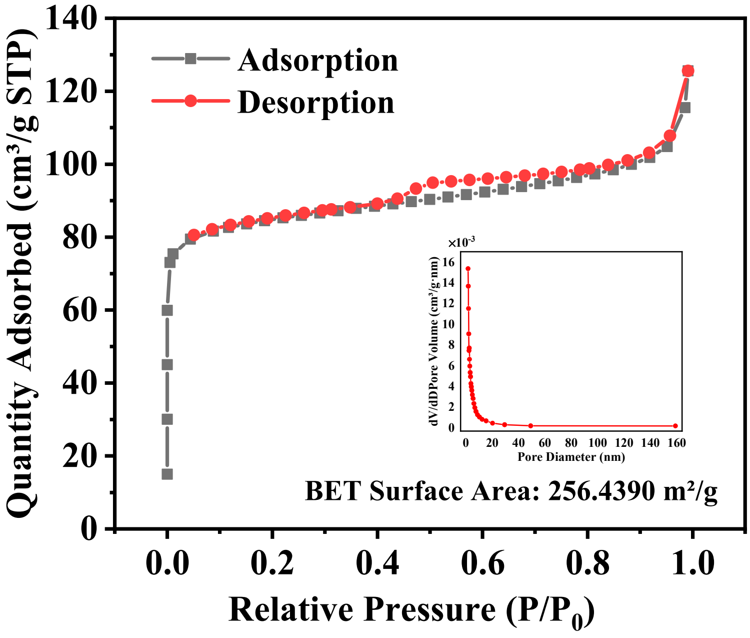

Figure S1. N_2 adsorption-desorption isotherm plot of Co-C/SiO_2.

Table S1. BET comparison between Co-C/SiO_2 and other catalysts.

	**BET specific surface area (m²/g)**	**Pore volume (cm³/g)**	**Pore size (nm)**	**k (min^{-1})**	**k-value ($\times 10^{-3} min^{-1}$)**	**Ref.**
Fe_3O_4@SiO_2@mSiO_2-Pd(0)	202.98	0.2419	20	/	/	[1]
NPC-800	676.9	0.86	/	0.0427	21.4	[2]
MNBC800	150.7	0.081	/	0.104	5.2	[3]
POP-1	33.39	/	15	/	/	[4]
Co-C/SiO_2	256.44	0.1942	3.0297	0.2271	28.4	This work

Table S2. Comparison of the binding energy and content of each element of Co-C/SiO_2 before and after the reaction.

		Binding Energy	Atomic (%)		Binding Energy	Atomic (%)
C 1s	Before reaction	284.4	91.1	After reaction	284.4	87.6
N 1s		399.11	1.65		399.29	2.06
O 1s		533.71	5.94		532.39	8.87
Si 2p		102.94	1.01		102.57	0.71
Co 2p1		802.67	0.3		795.49	0.54
Co 2p3		776.01	0		780.01	0.21

Table S3. Comparison between Co-C/SiO_2 and other Fenton-like catalysts for $^1O_2$ yield.

**Catalyst**	**Trapping reagent**	**$^1O_2$ yield**	**Ref.**
S-OMC-600	DPBF	4177.78 μmol/(g•L)	[5]
Ru_n/NC-850	DPBF	k-$^1O_2$ = 0.2954 min^{-1}	[6]
BvBN/Co	DPA	67.7 μM	[7]
Co-C/SiO_2	DPA	31.3 μM	This work

References

[1] Li, W.; Zhang, B.; Li, X.; Zhang, H. and Zhang, Q. Preparation and characterization of novel immobilized Fe_3O_4@SiO_2@mSiO_2–Pd(0) catalyst with large pore-size mesoporous for Suzuki coupling reaction. *Applied Catalysis A: General* **2013**, *459*, 65-72.

[2] Ma, W.; Du, Y.; Wang, N. and Miao, P. ZIF-8 derived nitrogen-doped porous carbon as metal-free catalyst of peroxymonosulfate activation. *Environmental Science and Pollution Research* **2017**, *24*, 16276–16288.

[3] Liu, C.; Chen, L.; Ding, D. and Cai, T. From rice straw to magnetically recoverable nitrogen doped biochar: Efficient activation of peroxymonosulfate for the degradation of metolachlor. *Appl. Catal. B Environ.* **2019**, *254*, 312-320.

[4] Liu, P.; Xing, L.; Lin, H.; Wang, H.; Zhou, Z. and Su, Z. Construction of porous covalent organic polymer as photocatalysts for RhB degradation under visible light. *Science Bulletin* **2017**, *62*, 931-937.

[5] Liu, S.; Lai, C.; Zhou, X.; Zhang, C.; Chen, L.; Yan, H.; Qin, L.; Huang, D.; Ye, H.; Chen, W.; Li, L.; Zhang, M.; Tang, L.; Xu, F. and Ma, D. Peroxydisulfate activation by sulfur-doped ordered mesoporous carbon: Insight into the intrinsic relationship between defects and $^1O_2$ generation. *Water Res.* **2022**, *221*, 118797.

[6] Bi, G.; Ding, R.; Song, J.; Luo, M.; Zhang, H.; Liu, M.; Huang, D. and Mu, Y. Discriminating the Active Ru Species Towards the Selective Generation of Singlet Oxygen from Peroxymonosulfate: Nanoparticles Surpass Single-Atom Catalysts. *Angewandte Chemie International Edition* **2024**, *63*, e202401551.

[7] Zhen, J.; Sun, J.; Xu, X.; Wu, Z.; Song, W.; Ying, Y.; Liang, S.; Miao, L.; Cao, J.; Lv, W.; Song, C.; Yao, Y. and Xing, M. M−N_3 Configuration on Boron Nitride Boosts Singlet Oxygen Generation via Peroxymonosulfate Activation for Selective Oxidation. *Angewandte Chemie International Edition* **2024**, *n/a*, e202402669.
